# Cyst Reduction by Melatonin in a Novel *Drosophila* Model of Polycystic Kidney Disease

**DOI:** 10.3390/molecules25225477

**Published:** 2020-11-23

**Authors:** Cassandra Millet-Boureima, Roman Rozencwaig, Felix Polyak, Chiara Gamberi

**Affiliations:** 1 Biology Department, Concordia University, Montreal, QC H4B 1R6, Canada; cassandra.millet@mail.concordia.ca; 2 BH Bioscience, Montreal, QC H3W 2L2, Canada; rachmilr@gmail.com (R.R.); rcq003@gmail.com (F.P.)

**Keywords:** polycystic kidney disease, renal cysts, melatonin, *Drosophila*, longevity, oxidative stress

## Abstract

Autosomal dominant polycystic kidney disease (ADPKD) causes progressive cystic degeneration of the renal tubules, the nephrons, eventually severely compromising kidney function. ADPKD is incurable, with half of the patients eventually needing renal replacement. Treatments for ADPKD patients are limited and new effective therapeutics are needed. Melatonin, a central metabolic regulator conserved across all life kingdoms, exhibits oncostatic and oncoprotective activity and no detected toxicity. Here, we used the *Bicaudal C* (*BicC*) *Drosophila* model of polycystic kidney disease to test the cyst-reducing potential of melatonin. Significant cyst reduction was found in the renal (Malpighian) tubules upon melatonin administration and suggest mechanistic sophistication. Similar to vertebrate PKD, the *BicC* fly PKD model responds to the antiproliferative drugs rapamycin and mimics of the second mitochondria-derived activator of caspases (Smac). Melatonin appears to be a new cyst-reducing molecule with attractive properties as a potential candidate for PKD treatment.

## 1. Introduction

### 1.1. Polycystic Kidney Disease 

Autosomal dominant polycystic kidney disease (ADPKD) is a genetic disease affecting 12.5 million people globally. ADPKD typically causes the progressive formation of cysts all along the renal tubules called nephrons [1]. ADPKD has been linked to mutations in the *PKD1* gene in about 80% of the cases and in *PKD2* in about 15% of patients, with the residual ~5% of cases remaining genetically unknown or linked to rare mutations in other loci [1]. Renal cystic disease in general, and ADPKD particularly, display complex metabolic alterations in the tissue of the renal tubule [2] as well as impaired fluid transport [1]. As cysts progressively grow, they compress and damage the surrounding renal parenchyma, impairing the neighboring nephrons, both cystic and non-cystic. The ensuing reduced renal function eventually leads to renal failure in one patient out of two [1].

Cystic growth in ADPKD displays some neoplastic characteristics [3,4]. Among several cancer pathways found to be dysregulated in ADPKD [5,6,7,8,9,10,11,12,13,14,15,16], activation of the mechanistic/mammalian target of rapamycin (mTOR) pathway was found to contribute to renal cyst cell proliferation in patients and ADPKD animal models [6,17,18,19,20,21]. As well, *myc*, a common oncogene elevated in cancer cells, was found to be upregulated in ADPKD [21,22,23]. Murine ADPKD models have helped to define the stages of disease progression, as well as study the effects of mutations [24]. These studies clearly indicated that the genetics of ADPKD-type cyst formation is complex. The discovery that human *PKD1* and its murine ortholog affect the expression of the gene *BICAUDAL C*, which in turn regulates MYC and mTOR has allowed to place *BICAUDAL C* genetically downstream of *PKD1* and upstream of *MYC* [21]. Originally discovered in the fruit fly *Drosophila melanogaster* ovary, the *Bicaudal C* gene encodes for the prototype of a family of RNA binding proteins (reviewed in [25]). The human *BICAUDAL C* gene is abbreviated as *BICC1*, the murine one as *Bicc1*, and the *Drosophila* one as *BicC*. Adult *BicC* mutant flies display key ADPKD features [21]. In ADPKD patients, cysts occur most frequently in the intermediate and terminal (distal) regions of the renal tubule [1]. Similarly, in the *BicC* mutant flies, cysts occur most frequently in the intermediate and terminal tubules [21]. Cyst occurrence in the terminal tubules is thought to disrupt fluid readsorption and urine concentration that normally occur in this region [1]. Similar to ADPKD, *BicC* mutant flies also display TOR activation and *myc* upregulation [21]. *Drosophila* has thus joined the arsenal of PKD-type cyst models to study the genetic underpinning of renal cystogenesis [21]. The fly features conservation of 75% of genes and pathways involved in human disease [26]. Renal pathway components are also highly conserved (reviewed in [27,28]). The *Drosophila* aglomerular renal system contains four anatomically separate renal (Malpighian) tubules analogous to the tubular regions of the human nephron. Reminiscent of nephron diversity in the human kidneys, each fly contains one pair of longer Malpighian tubules extending anteriorly and one pair of shorter ones oriented posteriorly. Such anterior and posterior pairs have distinct transcriptomes and functions [29]. Also similar to the human nephron, each fly Malpighian tubule displays functionally distinct regions with selective transport. In the fly, these are called proximal, intermediate, and terminal (reviewed in [27]). Unlike the human nephron, the *Drosophila* Malpighian tubules can be precisely dissected and examined to evaluate cyst-reducing molecules [27,30,31]. 

A mimetic of the second mitochondria-derived activator of caspases (Smac) was shown to ameliorate cysts in a murine ADPKD model [32]. Using the *Drosophila BicC* PKD model, we have recently characterized the substantial cyst-reducing properties of four novel Smac mimetics confirming a potential for Smac mimicry in ameliorating PKD, which further underscores conservation of the renal cystic mechanisms [30]. ADPKD is incurable, urging the discovery of anti-cystic drugs. A repurposed antagonist of the vasopressin V2 receptor, tolvaptan has been approved for use in a subset of ADPKD patients between the ages of 18 and 50, presenting a moderate decline in renal function, as well as fast disease progression [33,34]. Tolvaptan-induced hepatotoxicity and possible loss of efficacy over time limit its broad use in PKD therapy [34]. In an effort to offer remedy to the larger ADPKD patient population, several molecules and diet-induced strategies targeting altered cystic cell metabolism are being studied (e.g., [35,36,37]). Unlike several antineoplastic compounds, such molecules exhibit low general toxicity and may present wider applicability. In fact, because ADPKD is chronic and must be managed in the long-term, an ideal ADPKD drug would have no to low toxicity.

### 1.2. Melatonin 

Melatonin (*N*-acetyl-5-methoxytrypamine) is a ubiquitous neurohormone that, in humans, is produced and secreted at night from the pineal gland and is also made locally by cells in the brain [38,39], skin [40], gastrointestinal tract [41], lymphocytes [42], several other tissues, and potentially all cells [43]. Melatonin is thought to function pleiotropically to synchronize most physiological functions with the circadian cycle (reviewed in [44]). In mammalians, melatonin acts via G protein-coupled receptors MT1 and MT2, which are ubiquitously expressed in the central nervous system [45,46,47,48,49]. Other melatonin receptors may also exist, and melatonin may also function in receptor-independent ways (reviewed in [50]). Melatonin reduces oxidative stress, anxiety, hypertension [51,52,53] and pain [54]. Melatonin has been found to extend the lifespan of rodents and fruit flies, while treating age-related diseases such as premature aging and carcinogenesis [49,53,55,56,57,58,59].

Consistent with its numerous physiological effects, melatonin has long been known as an oncostatic in a variety of cancer types and is thought to function at multiple levels ([58]; reviewed in [60,61]). Melatonin reduces proliferation of several cancer cells in vitro, e.g., breast [62,63,64,65,66,67,68], melanoma [69,70] via several growth factor pathways (e.g., insulin, TOR, mitogen activated protein kinases, MAPK, epithelial growth factor, EGF [71,72,73,74,75,76]; reviewed in [61]) and regulates energy production via the insulin pathway, nutrient uptake and glycolysis (ib.). Melatonin exerts anti-genotoxic, anti-mutagenic and anti-oxidative effects in vitro and in vivo, while bringing reactive oxidative species to toxic levels specifically in cancer cells [56,58,77,78,79,80,81,82,83,84,85,86,87,88,89,90,91]. Simultaneously, melatonin can induce cancer cell apoptosis and cell death via multiple pathways [51,75,92,93,94]. Moreover, melatonin was found to suppress angiogenesis by inhibiting the abnormal proliferation and migration of endothelial cells ([68,95,96,97]; reviewed in [98]) and to play immunomodulatory functions (reviewed in [61]). One notable ADPKD feature is the hyperproliferation of the tubular epithelium to form cysts through the activation of evolutionarily conserved pathways ([3,4,99], reviewed in [16]). Moreover, ADPKD causes oxidative stress and inflammation [100,101,102]. The cellular pathways of reactive oxidative response are conserved in *Drosophila* [103,104,105,106].

Considering the wide and potentially beneficial effects of melatonin, as well as its low toxicity profile [107,108,109], we tested the cyst-reducing potential of melatonin utilizing the *Drosophila* PKD model. The *BicC* fly model of renal cystogenesis was previously used successfully to test the anti-cystic activity of rapamycin [21] and Smac mimetics [30]. Here, we report that melatonin was found to substantially reduce cysts in the *Drosophila* PKD model.

## 2. Results

### 2.1. Melatonin Significantly Reduced Cysts in the Renal Tubule of BicC^Δ/YC33^ Mutants 

**Figure 1:**
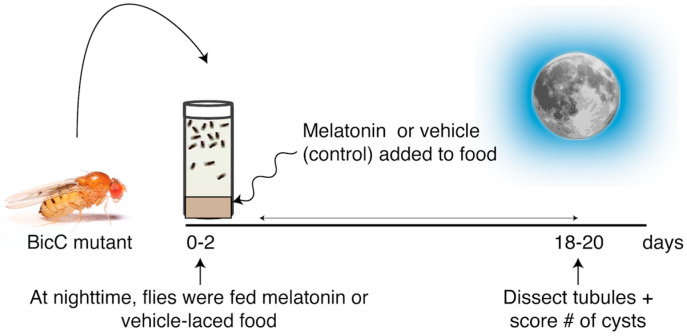
Protocol for testing the melatonin anti-cystic activity. *BicC^Δ/YC33^* and *BicC^Δ/IIF34^* flies (aged 0–2d) were placed in food-containing vials mixed with either vehicle (ethanol) or 150 µM melatonin at nighttime. Malpighian tubules were micro-dissected after 18 days of treatment and cysts scored (population aged 18–20d).

**Figure 2:**
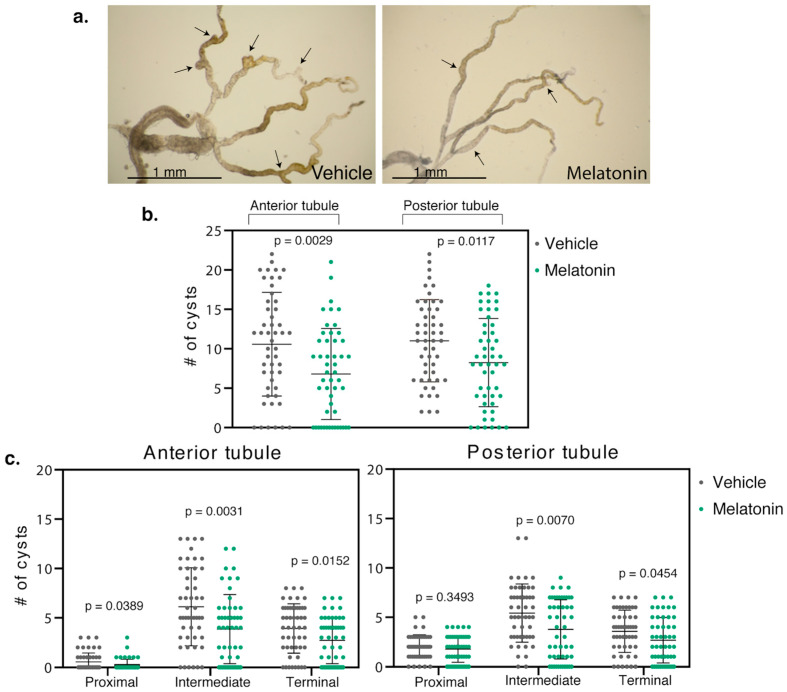
Melatonin reduced cysts in the renal tubule of *BicC^Δ/YC33^* flies. (**a**) Representative Malpighian tubules micro-dissected from *BicC^Δ/YC33^* flies treated with either vehicle (ethanol) or 150 µM melatonin were photographed ex vivo. Arrows indicate exemplary cysts. (**b**) Nested plots reporting overall number of cysts found in each anterior and posterior tubule pair of 50 vehicle- and 50 melatonin-treated cystic flies, with mean and standard deviation. (**c**) Regional analyses. Nested plots indicating the number of cysts found in the proximal, intermediate, and terminal region of the anterior and posterior tubule pairs of the flies in b, with mean and standard deviation. *p* values (with Welch’s correction) are indicated. Treatments are shown with color: vehicle, left, grey; melatonin, right, green. Melatonin treatment significantly reduced cysts in all regions of the Malpighian tubules of *BicC^Δ/YC33^* flies (*p* < 0.05), except for the proximal region in the posterior tubules.

Populations of *BicC^Δ/YC33^* flies aged 0–2 days were fed either vehicle (ethanol) or 150 µM melatonin and treated in parallel (Figure 1). When compared to the vehicle-treated siblings, 150 µM melatonin significantly reduced cysts in both the anterior and posterior tubules of the milder *BicC^Δ/YC33^* flies (*n* = 50) by 36% (total 529 vs. 340 cysts, *p* = 0.0029) and 31% (total 551 vs. 412 cysts, *p* = 0.0117), respectively (Table 1, Figure 2).

### 2.2. Melatonin Treatment Displayed Regional Specificity in BicC^Δ/YC33^ Mutants

Similar to human nephrons, the *Drosophila* renal tubules display regional specialization and differential response to Smac mimics [30]. Thus, we examined the regional physiological response to melatonin treatment. In the *BicC^Δ/YC33^* flies, melatonin appeared to reduce cysts in the proximal, intermediate, and terminal regions of the anterior tubules, respectively, by 59, 37 and 31% (total 27 vs. 11 cysts (*p* = 0.0389), 306 vs. 193 cysts (*p* = 0.0031), and 196 vs. 136 cysts (*p* = 0.0152)) (Table 2, Figure 2). In the posterior tubules, melatonin administration diminished cysts in the proximal, intermediate and terminal regions, respectively, by 12, 30 and 25% (total 101 vs. 89 cysts (*p* = 0.3493), 271 vs. 189 cysts (*p* = 0.0070), and 179 vs. 134 cysts (*p* = 0.0454)) (Table 2, Figure 2).

Therefore, melatonin administration significantly reduced cysts in *BicC^Δ/YC33^* flies overall, with apparent slightly higher efficacy in the anterior tubule. Moreover, melatonin reduced cysts in the proximal, intermediate and terminal regions of the anterior tubules and in the intermediate and terminal regions of the posterior tubules, with the proximal region displaying a trend toward cyst reduction. 

### 2.3. Melatonin Treatment Was Less Efficient in BicC^Δ/IIF34^ Mutants

**Figure 3:**
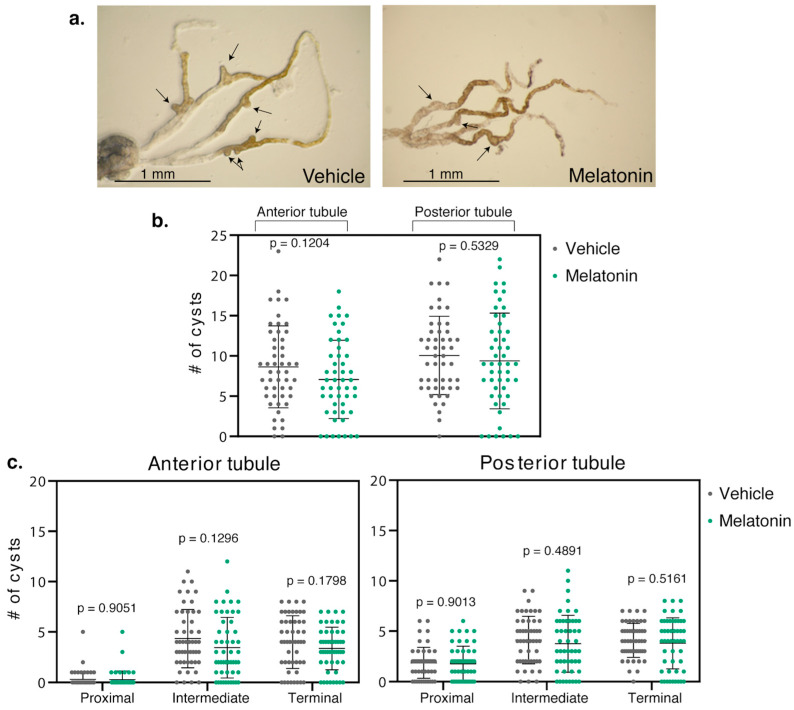
Melatonin did not reduce cysts in *BicC^Δ/IIF34^* flies. (**a**) Representative Malpighian tubules micro-dissected from *BicC^Δ/IIF34^* flies treated with either vehicle (ethanol) or 150 µM melatonin were photographed ex vivo. Arrows indicate exemplary cysts. (**b**) Nested plots reporting overall number of cysts found in each anterior and posterior tubule pair of 50 vehicle- and 50 melatonin-treated cystic flies, with mean and standard deviation. (**c**) Regional analyses. Nested plots indicating the number of cysts found in the proximal, intermediate, and terminal region of the anterior and posterior tubule pairs of the flies in b, with mean and standard deviation. *p* values (with Welch’s correction) are indicated. Treatments are color-coded: vehicle, left, grey; melatonin, right, green. Melatonin treatment of *BicC^Δ/IIF34^* flies produced a trend in cyst reduction, however, did not significantly differentiate the treated vs. untreated populations.

The *BicC* allelic combination, *BicC^Δ/IIF34^* yields more severe defects than *BicC^Δ/YC33^* despite expressing higher levels of a truncated BicC protein [110] and may be a dominant negative [21]. Melatonin was administered to the *BicC^Δ/IIF34^* flies (n = 50) with identical procedure. Melatonin induced an overall trend towards reduced cysts in both anterior and posterior tubules. However, phenotypic variability was such that *p*-values resulted above significance threshold. Specifically, compared to vehicle-treatment, the anterior tubule displayed 18% less cysts (total 432 vs. 354 cysts, *p* = 0.1204) and the posterior tubule 7% less (total 503 vs. 469 cysts, *p* = 0.5329) (Table 1, Figure 3). Along the different regions of the Malpighian tubules of *BicC^Δ/IIF34^* flies, melatonin administration also produced a trend in cyst-reduction in the proximal, intermediate, and terminal regions of the anterior tubules, respectively, by 7, 21 and 16% (total 15 vs. 14 cysts (*p* = 0.9051), 217 vs. 172 cysts (*p* = 0.1296), and 200 vs. 168 cysts (*p* = 0.1798)) (Table 2, Figure 3). In the posterior tubules, melatonin diminished cysts in the proximal, intermediate and terminal regions by 2, 9 and 7% (total 93 vs. 91 cysts (*p* = 0.9013), 206 vs. 188 cysts (*p* = 0.4891), and 204 vs. 190 cysts (*p* = 0.5161)) (Table 2, Figure 3). Therefore, melatonin administration yielded a trend in cyst reduction in the more severely cystic *BicC^Δ/IIF34^* flies; however, *p*-values remained above significance threshold.

## 3. Discussion

Melatonin, a pleiotropic hormone, has long been studied for the treatment of age-related diseases and carcinogenesis. Suggesting a renoprotective role, decreased melatonin levels correlate with renal dysfunction in chronic kidney disease (CKD) [111,112,113,114]. Melatonin was found to protect rat kidneys against oxidative damage [115,116,117]. Upon carbon tetrachloride-induced oxidative damage, melatonin restored antioxidant enzyme levels and improved kidney histopathology [116]. Notably, diabetic and IgA nephropathies are characterized by increased circulating reactive oxygen species [118,119]. Early ADPKD pathogenesis has a strong component of oxidative stress [120] with reduced expression of antioxidant enzymes [100]. The reactive oxidative response is conserved in *Drosophila* [103,104,105,106,121,122,123]. Therefore, melatonin by lowering oxidative damage to the renal tubular cells, may similarly improve cysts in both mammalian and *Drosophila* renal tubules. Here, we report that melatonin exhibits cyst-reducing effects in the first-in-kind *Drosophila* model of PKD [21,27,30,31]. 

The *BicC* PKD fly model recapitulates phenotypic and molecular hallmarks of *PKD1*-induced PKD [21] and conserved pharmacological response to Smac mimetics [30]. In the mammalian response to pro-apoptotic signals and TNF-α/TNF receptor (TNFR) activation, the Smac protein is released from the mitochondria, which activates the caspase cascade [124]. Smac mimicry has been exploited in oncology to induce apoptosis in TNF-α-dependent cancers [125,126]. Administration of small peptide mimetics of the Smac has been shown to be sufficient to activate the caspase cascade and mitigate cancer [125,126]. Both ADPKD patients and the *Pkd1^−/−^* mouse display high TNF-α amounts in the cystic fluid; moreover, the cystic cells feature higher-than-normal expression of the TNFR1 receptor. Unlike other tubular cells, the cyst-lining cells have abundant TNFR and are bathed in TNF-α-rich fluid, which is thought to fuel an autoactivating loop promoting cyst growth [32,127]. Because of such specific and constitutive TNF-α activation in the cells of ADPKD cysts, one Smac mimetic has been tested in a murine model to preferentially eliminate cystic cells while sparing the non-cystic tubular portions [32]. TNF signaling is highly conserved in *Drosophila* [128,129,130,131]. We showed that, similar to the ADPKD mouse, administration of four novel Smac mimetics to the *BicC* fly PKD model significantly reduced cysts in the renal tubules [30]. This underscores the conservation of cystic pathways between human and *Drosophila*. Interestingly, melatonin has been shown to decrease TNF-α expression [132,133,134,135], which raises the possibility that melatonin pleiotropic functions may contribute to renal tubule normalization. In further support of this possibility, melatonin is a known antiproliferative that normalizes several overactive pathways in both cancer and ADPKD, e.g., ERK, mTOR, PI3K/Akt, PKC [1,16,53,58,71,136,137,138,139,140,141,142,143]. Note, the *BicC* fly model of PKD also exhibits hyperactive mTOR [21].

In 1861, it was reported that renal physiology has circadian rhythmicity [144]. Excretion of water, urea and electrolytes all follow 24-h cyclicity [145,146]. Defective glomerular filtration in patients with CKD may disturb sleep (reviewed in [113]). In end-stage renal disease (ESRD), the severity of insomnia appeared to correlate directly with disease progression [112], and inversely with melatonin levels [147,148,149,150,151,152,153]. While specific knowledge of the ADPKD situation is limited, deteriorating kidney function reduces circadian rhythm amplitude [112], suggesting that patients in late stage ADPKD may also have reduced melatonin level and/or function. Melatonin has only been tested in two small clinical trials to treat CKD-related sleep disturbances and the results of one such trials have been published [154]. Small trial NCT04336566 was designed to test melatonin effects on renal function in CKD, however, its results have not been made public. Melatonin pre-treatment has also been found to potentiate the beneficial anti-apoptosis, anti-oxidation, anti-inflammation effects of mesenchymal stem cell (MSC) therapy to treat acute kidney injury (AKI) and CKD ([155], reviewed in [156]). In a rat CKD model, melatonin pre-treated MSCs also reduced fibrosis in the kidney [135,156]. Together, this evidence strongly suggests that melatonin is crucial for maintaining proper kidney function and that nephrological diseases compromising renal capacity appear to upset the melatonin-dependent pathways.

In the *Drosophila* PKD model, nightly administration of 150 µM melatonin efficiently reduced cysts in the *BicC^Δ/YC33^* mutants, compared to vehicle-treated flies. These effects were observed both along the entire Malpighian tubules, and regionally. Melatonin reduced cysts in the anterior and posterior tubules by over 30% (*p* < 0.012). In the terminal, intermediate and proximal regions of the anterior tubules of *BicC^Δ/YC33^* flies, melatonin reduced cysts by 31–59% (*p* < 0.039). In the posterior tubules, melatonin treatment reduced cysts in the terminal and intermediate regions by 25 and 30% respectively (*p* < 0.045), while showing a trend towards reduction in the proximal region. Such differential response to melatonin is expected to be rooted onto the functional and physiological differences documented for the anterior and posterior tubules and the tubule regional specializations ([157]; reviewed in [27]). This property is also consistent with our previous observations of regionally distinct effects of Smac mimetics in the same PKD fly model [30]. In contrast to the *BicC^Δ/YC33^* mutants in which melatonin significantly reduced renal cysts, *BicC^Δ/IIF34^* flies only showed a trend of regional cyst reduction. Notably, the *BicC^Δ/IIF34^* genotype may be dominant negative [21]. This suggests that the genotype may influence the extent of melatonin response of the cystic renal tubule. Future investigations will decipher how melatonin may reduce renal cysts and how specific *BicC* mutations may affect the melatonin cyst-reducing activity in the renal tubule. 

Several core cellular pathways are disrupted in ADPKD. As disease progresses, physiological compensation (e.g., through vasopressin signaling) compounds cellular changes [1]. The molecular detail of PKD pathology is largely unknown [24]. Melatonin biological activity as a potential ameliorator of PKD cystic pathology is intriguing. Firstly, its low toxicity is especially attractive for the long-term management of chronic PKD. Second, melatonin is a master cellular regulator conserved throughout evolution with pleiotropic functions that may help normalize several dysregulated pathways in PKD, e.g., oxidative stress, cell proliferation, fibrosis, renal circadian functions. Tolvaptan, a vasopressin V2 receptor antagonist, appears to primarily target vasopressin-dependent compensation in PKD through mechanisms conserved among mammals. Melatonin may potentially be combined with tolvaptan to reduce toxicity and treat PKD. In oncology, melatonin has been found to potentiate several chemotherapeutics, while simultaneously protecting the patient from their ill effects (e.g., [158,159,160,161,162,163,164,165,166,167,168]). If such property is conserved to its cyst-reducing activity, melatonin may become a prospective candidate for utilization as a single or combination drug in PKD therapy.

## 4. Materials and Methods 

### 4.1. Fly Lines and Husbandry 

*BicC* mutants were generated as in [21,30]. Briefly, virgin female flies harboring a *BicC* deletion in trans to the *CyO* balancer chromosome, *Df(2L)RA5*/*CyO* (obtained from the Bloomington *Drosophila* Stock Center) were crossed with males carrying one of two hypomorphic *BicC* mutations in trans to *CyO*, namely *BicC^YC33^/CyO* and *BicC^IIF34^/CyO*. *BicC* mutants were selected as the straight-winged progeny with genotypes *Df(2L)RA5*/*BicC^YC33^* (hereby *BicC^Δ/YC33^*) and *Df(2L)RA5*/*BicC^IIF34^*, (hereby *BicC^Δ/IIF34^*). Such *BicC* allelic combinations produce truncated proteins and sterile *BicC* flies [21]. The *BicC^Δ/IIF34^* genotype yields a more severe cystic phenotype than the *BicC^Δ/YC33^* combination and may be dominant negative [21]. Eclosed adult flies were collected every two days to generate 0–2-day old populations to be used in the assays.

### 4.2. Cystic Index

The cystic analysis was performed as in [30], with the following modifications. The 0–2-day old *BicC* mutant females were housed in vials containing 2 mL cornmeal food (Jazzmix, Fisher Scientific) that were replaced every three days to ensure freshness. During daytime, flies were fed plain cornmeal food. In the evening, flies were transferred into identical vials to which equal volumes (50 µL) of either vehicle (ethanol, control) or 150 µM melatonin (resuspended in ethanol) were added and then incubated overnight. In the morning, flies were transferred to vials with plain cornmeal food. The timing of dissection of the Malpighian tubules for melatonin efficacy was determined empirically with ten female flies micro-dissected at 8, 12, 18, and 25 days after treatment beginning and 18 days post-treatment was chosen for further analyses. This corresponds to fly populations of individuals aged 18–20 days. Larger 0–2-day old fly populations were then established and fed either melatonin or vehicle as above for 18 days, after which the Malpighian tubules were micro-dissected from 50 female flies in phosphate buffered saline (PBS). Cysts were counted separately for the anterior and posterior tubules, differentiating each tubular region (i.e., proximal, intermediate and terminal), due to their known physiological differences ([157]; reviewed in [27]). Wild-type tubules are elongated and regularly shaped, while *BicC* mutant tubules appear larger and deformed by cysts. Cysts were scored as any tubular deformation creating uni- or bi-lateral expansions or extra-branches as in [30]. To determine that flies ingested melatonin, green dye was added to the food and melatonin mixture. After three days, the dye can be visualized through the semi-transparent abdominal cuticle (Figure S1). Data were plotted using the Graphpad Prism 8.0 software (https://www.graphpad.com/scientific-software/prism/) as nested distributions and analyzed statistically. Unpaired t-tests were performed with both Excel and Graphpad Prism 8.0 and the Welch’s correction added, because the populations may not have equal standard deviations. *p* values of less than 0.05 were considered significant and indicative of cyst-reducing activity. The cystic index raw data can be found in Table S1. 

### 4.3. Microscopy

Malpighian tubules from aged and treated flies as indicated were manually micro-dissected in 1× PBS, washed and equilibrated into a 3:1 1× PBS:glycerol solution as in [30] and photographed on a Leica MZ FLIII Fluorescence Stereomicroscope with Leica MZ series 10×/21B Widefield adjustable eyepieces equipped with a Canon DS126201 EOS 5D MARK II camera, using visible light. Canon raw files (CR2) were converted into TIF format using the Adobe Lightroom 3.2 software (Adobe Systems, San Jose, CA, USA).

## Figures and Tables

**Table 1:** Overall cyst reduction upon melatonin treatment of *BicC* mutants.

	Anterior Tubules	Posterior Tubules
*BicC^Δ/YC33^* (*n* = 50)	**36%** (*p* = 0.0029)	**31%** (*p* = 0.017)
*BicC^Δ/IIF34^* (*n* = 50)	*18%* (*n.s.*)	*7%* (*n.s.*)

Bold indicates significant cyst reduction; Italics indicate populations yielding *p* > 0.05 (statistically not significant, *n.s*.).

**Table 2:** Regional cyst reduction upon melatonin treatment of *BicC* mutants.

	Anterior Tubules			Posterior Tubules		
	Proximal	Intermediate	Terminal	Proximal	Intermediate	Terminal
*BicC^Δ/YC33^*	**59%** *p* = 0.0389	**37%** *p* = 0.0031	**31%** *p* = 0.0152	*12%* *p = 0.3493*	**30%** *p* = 0.0070	**25%** *p* = 0.0454
*BicC^Δ/IIF34^*	*7%* *p = 0.9051*	*21%* *p = 1296*	*16%* *p = 0.1798*	*2%* *p = 0.9013*	*9%* *p = 0.4891*	*7%* *p = 0.5161*

Bold indicates significant cyst reduction; Italics indicate populations yielding *p* > 0.05 (statistically not significant).

## Supplementary Materials

The following are available online, Figure S1: Melatonin feeding control, Table S1: Cystic index raw data.
