# Supplementary material for: Cyst Reduction by Melatonin in a Novel Drosophila Model of Polycystic Kidney Disease
Source: Molecules. 2020 Nov 23;25(22):5477. doi: 10.3390/molecules25225477 (PMC7700119; doi:10.3390/molecules25225477)
Supplement: Supplementary file 1 [file molecules-25-05477-s001.pdf]

# Cyst reduction by melatonin in a novel *Drosophila* model of polycystic kidney disease

**Cassandra Millet-Boureima [1], Roman Rozencwaig [2], Felix Polyak [2] and Chiara Gamberi [1,*]**

[1] Biology Department, Concordia University, Montreal H4B 1R6, QC, Canada; cassandra.millet@mail.concordia.ca (C.M.B.)
[2] BH Bioscience, Montreal, H3W 2L2; rachmilr@gmail.com (R.R.) ; rcq003@gmail.com (F.P.)
* Correspondence: chiara.gamberi@concordia.ca (C.G.); Tel.:+1-524 848 2424 ext. 3395

***BicC*** $^{\Delta/YC33}$

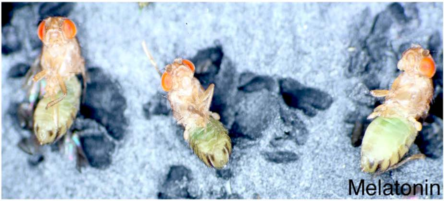


***BicC*** $^{\Delta/IIF34}$

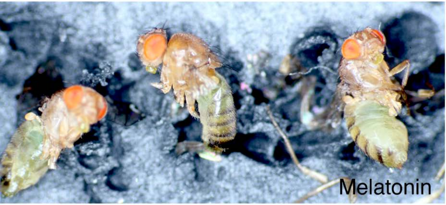


**Figure S1. Melatonin feeding control.** *BicC* flies fed melatonin mixed with green dye for three days, displayed ingested food contents visible through their semi-transparent abdominal cuticle. Legs were clipped for better visualization.

## *BicC* [*delta/YC33* ] 18-20d (18 days after treatment)

| CONTROL | Anterior Terminal | Anterior Intermediate | Anterior Proximal | Posterior Terminal | Posterior Intermediate | Posterior Proximal |
|---|---|---|---|---|---|---|
| 1 | 2 | 6 | 2 | 4 | 3 | 0 |
| 2 | 7 | 10 | 0 | 5 | 8 | 3 |
| 3 | 6 | 5 | 0 | 5 | 6 | 3 |
| 4 | 5 | 13 | 1 | 5 | 9 | 5 |
| 5 | 5 | 9 | 2 | 4 | 7 | 1 |
| 6 | 6 | 13 | 1 | 4 | 7 | 1 |
| 7 | 8 | 13 | 1 | 6 | 8 | 3 |
| 8 | 5 | 10 | 0 | 7 | 8 | 5 |
| 9 | 6 | 12 | 0 | 4 | 5 | 1 |
| 10 | 4 | 7 | 1 | 3 | 7 | 2 |
| 11 | 7 | 11 | 2 | 4 | 9 | 3 |
| 12 | 8 | 10 | 3 | 3 | 8 | 2 |
| 13 | 7 | 11 | 1 | 6 | 9 | 3 |
| 14 | 6 | 6 | 0 | 6 | 5 | 1 |
| 15 | 6 | 11 | 3 | 7 | 8 | 1 |
| 16 | 6 | 7 | 1 | 6 | 13 | 3 |
| 17 | 4 | 8 | 0 | 6 | 13 | 2 |
| 18 | 6 | 7 | 0 | 5 | 7 | 2 |
| 19 | 5 | 9 | 2 | 3 | 6 | 4 |
| 20 | 6 | 7 | 2 | 6 | 5 | 4 |
| 21 | 5 | 5 | 3 | 5 | 4 | 0 |
| 22 | 4 | 8 | 0 | 3 | 6 | 2 |
| 23 | 6 | 13 | 0 | 7 | 9 | 2 |
| 24 | 2 | 10 | 0 | 6 | 7 | 3 |
| 25 | 8 | 11 | 1 | 7 | 7 | 1 |
| 26 | 0 | 3 | 0 | 3 | 3 | 3 |
| 27 | 7 | 4 | 1 | 4 | 7 | 2 |
| 28 | 5 | 3 | 0 | 4 | 5 | 2 |
| 29 | 2 | 5 | 0 | 4 | 8 | 3 |
| 30 | 0 | 0 | 0 | 1 | 1 | 2 |
| 31 | 6 | 7 | 0 | 3 | 5 | 1 |
| 32 | 3 | 5 | 0 | 4 | 6 | 0 |
| 33 | 3 | 2 | 0 | 4 | 7 | 2 |
| 34 | 3 | 3 | 0 | 2 | 3 | 1 |
| 35 | 1 | 2 | 0 | 0 | 3 | 1 |
| 36 | 4 | 5 | 0 | 0 | 3 | 3 |
| 37 | 2 | 2 | 0 | 3 | 2 | 1 |

| | | | | | | |
|---|---|---|---|---|---|---|
| 38 | 0 | 0 | 0 | 2 | 2 | 2 |
| 39 | 1 | 4 | 0 | 2 | 4 | 1 |
| 40 | 5 | 5 | 0 | 6 | 5 | 0 |
| 41 | 4 | 5 | 0 | 0 | 3 | 3 |
| 42 | 3 | 4 | 0 | 1 | 3 | 1 |
| 43 | 0 | 0 | 0 | 0 | 2 | 0 |
| 44 | 0 | 0 | 0 | 1 | 1 | 2 |
| 45 | 3 | 5 | 0 | 2 | 3 | 3 |
| 46 | 0 | 0 | 0 | 0 | 0 | 2 |
| 47 | 2 | 2 | 0 | 2 | 4 | 2 |
| 48 | 2 | 5 | 0 | 1 | 2 | 2 |
| 49 | 0 | 0 | 0 | 0 | 0 | 2 |
| 50 | 0 | 3 | 0 | 3 | 5 | 3 |
| Total # of cys | 196 | 306 | 27 | 179 | 271 | 101 |

| MELATONIN | Anterior Terminal | Anterior Intermediate | Anterior Proximal | Posterior Terminal |
|---|---|---|---|---|
| 1 | 4 | 5 | 0 | 1 |
| 2 | 6 | 12 | 3 | 4 |
| 3 | 2 | 5 | 0 | 2 |
| 4 | 5 | 8 | 2 | 6 |
| 5 | 4 | 5 | 0 | 2 |
| 6 | 5 | 6 | 0 | 1 |
| 7 | 3 | 9 | 1 | 5 |
| 8 | 4 | 10 | 1 | 6 |
| 9 | 4 | 4 | 0 | 4 |
| 10 | 0 | 0 | 0 | 3 |
| 11 | 5 | 6 | 0 | 1 |
| 12 | 3 | 5 | 1 | 7 |
| 13 | 7 | 5 | 0 | 7 |
| 14 | 6 | 10 | 0 | 6 |
| 15 | 0 | 0 | 0 | 0 |
| 16 | 5 | 4 | 0 | 5 |
| 17 | 4 | 1 | 0 | 3 |
| 18 | 7 | 12 | 0 | 7 |
| 19 | 4 | 9 | 0 | 6 |
| 20 | 6 | 9 | 0 | 5 |
| 21 | 7 | 4 | 1 | 5 |
| 22 | 4 | 2 | 1 | 5 |
| 23 | 5 | 6 | 0 | 3 |
| 24 | 0 | 0 | 0 | 0 |
| 25 | 5 | 7 | 0 | 6 |
| 26 | 0 | 0 | 0 | 0 |
| 27 | 3 | 6 | 0 | 2 |
| 28 | 3 | 5 | 0 | 0 |
| 29 | 5 | 6 | 0 | 2 |
| 30 | 2 | 2 | 0 | 4 |
| 31 | 0 | 0 | 0 | 0 |
| 32 | 0 | 5 | 1 | 4 |
| 33 | 4 | 2 | 0 | 1 |
| 34 | 1 | 4 | 0 | 2 |
| 35 | 0 | 0 | 0 | 0 |
| 36 | 4 | 2 | 0 | 2 |
| 37 | 2 | 1 | 0 | 1 |

| | | | | | | |
|---|---|---|---|---|---|---|
| | | 38 | 4 | 5 | 0 | 4 |
| | | 39 | 0 | 0 | 0 | 0 |
| | | 40 | 0 | 0 | 0 | 0 |
| | | 41 | 0 | 0 | 0 | 3 |
| | | 42 | 1 | 4 | 0 | 3 |
| | | 43 | 0 | 0 | 0 | 0 |
| | | 44 | 0 | 0 | 0 | 1 |
| | | 45 | 0 | 0 | 0 | 1 |
| | | 46 | 0 | 2 | 0 | 1 |
| | | 47 | 1 | 1 | 0 | 0 |
| | | 48 | 0 | 0 | 0 | 0 |
| | | 49 | 0 | 0 | 0 | 0 |
| | | 50 | 1 | 4 | 0 | 3 |
| 1080 | | Total # of cys | 136 | 193 | 11 | 134 |

| Posterior Intermediate | Posterior Proximal |
|---|---|
| 7 | 1 |
| 7 | 1 |
| 2 | 0 |
| 9 | 2 |
| 6 | 2 |
| 7 | 3 |
| 7 | 3 |
| 8 | 4 |
| 6 | 3 |
| 6 | 1 |
| 5 | 3 |
| 6 | 1 |
| 7 | 3 |
| 7 | 3 |
| 0 | 0 |
| 7 | 4 |
| 2 | 0 |
| 7 | 3 |
| 5 | 4 |
| 8 | 3 |
| 7 | 1 |
| 6 | 0 |
| 8 | 1 |
| 0 | 0 |
| 7 | 2 |
| 0 | 2 |
| 3 | 3 |
| 6 | 3 |
| 4 | 1 |
| 1 | 2 |
| 0 | 0 |
| 1 | 3 |
| 1 | 2 |
| 4 | 2 |
| 0 | 3 |
| 2 | 0 |
| 0 | 0 |

| | | |
|---|---|---|
| 5 | 2 | |
| 0 | 2 | |
| 0 | 1 | |
| 1 | 1 | |
| 2 | 4 | |
| 0 | 0 | |
| 1 | 2 | |
| 1 | 1 | |
| 3 | 4 | |
| 2 | 3 | |
| 0 | 0 | |
| 0 | 0 | |
| 5 | 0 | |
| 189 | 89 | 752 |

## ***BicC* [*delta/IIF34* ] 18-20d** (18 days of treatment)

| CONTROL | Anterior Terminal | Anterior Intermediate | Anterior Proximal | Posterior Terminal | Posterior Intermediate | Posterior Proximal |
|---|---|---|---|---|---|---|
| 1 | 7 | 8 | 0 | 7 | 7 | 5 |
| 2 | 3 | 3 | 0 | 5 | 6 | 1 |
| 3 | 8 | 6 | 0 | 5 | 4 | 2 |
| 4 | 2 | 10 | 0 | 5 | 4 | 3 |
| 5 | 6 | 2 | 1 | 6 | 6 | 2 |
| 6 | 6 | 7 | 1 | 6 | 7 | 4 |
| 7 | 7 | 5 | 0 | 5 | 5 | 2 |
| 8 | 7 | 10 | 0 | 5 | 7 | 2 |
| 9 | 7 | 7 | 0 | 5 | 5 | 6 |
| 10 | 5 | 4 | 2 | 5 | 6 | 2 |
| 11 | 8 | 5 | 0 | 4 | 7 | 3 |
| 12 | 6 | 7 | 0 | 6 | 5 | 1 |
| 13 | 8 | 9 | 1 | 7 | 9 | 6 |
| 14 | 5 | 6 | 0 | 3 | 5 | 1 |
| 15 | 7 | 11 | 5 | 7 | 9 | 3 |
| 16 | 8 | 9 | 0 | 7 | 7 | 5 |
| 17 | 7 | 9 | 1 | 6 | 6 | 4 |
| 18 | 2 | 1 | 1 | 4 | 3 | 0 |
| 19 | 0 | 7 | 0 | 2 | 3 | 2 |
| 20 | 0 | 5 | 0 | 3 | 4 | 2 |
| 21 | 2 | 8 | 0 | 3 | 2 | 0 |
| 22 | 6 | 7 | 0 | 4 | 4 | 2 |
| 23 | 2 | 4 | 1 | 5 | 8 | 3 |
| 24 | 4 | 3 | 0 | 6 | 5 | 2 |
| 25 | 0 | 2 | 0 | 3 | 5 | 1 |
| 26 | 4 | 4 | 0 | 4 | 5 | 2 |
| 27 | 6 | 3 | 0 | 3 | 7 | 2 |
| 28 | 4 | 3 | 0 | 3 | 2 | 0 |
| 29 | 3 | 2 | 0 | 5 | 6 | 2 |
| 30 | 0 | 1 | 0 | 1 | 1 | 1 |
| 31 | 0 | 0 | 0 | 0 | 0 | 0 |
| 32 | 0 | 3 | 1 | 4 | 1 | 2 |
| 33 | 4 | 5 | 0 | 3 | 4 | 2 |
| 34 | 2 | 3 | 0 | 3 | 2 | 2 |
| 35 | 1 | 0 | 0 | 4 | 2 | 0 |
| 36 | 4 | 4 | 0 | 2 | 5 | 0 |
| 37 | 5 | 4 | 0 | 6 | 4 | 2 |

| | | | | | | |
|---|---|---|---|---|---|---|
| 38 | 6 | 4 | 0 | 3 | 2 | 0 |
| 39 | 4 | 4 | 1 | 6 | 3 | 2 |
| 40 | 5 | 3 | 0 | 4 | 2 | 0 |
| 41 | 0 | 0 | 0 | 2 | 2 | 0 |
| 42 | 5 | 2 | 0 | 3 | 2 | 1 |
| 43 | 4 | 2 | 0 | 5 | 0 | 1 |
| 44 | 4 | 2 | 0 | 3 | 1 | 0 |
| 45 | 4 | 1 | 0 | 2 | 2 | 2 |
| 46 | 2 | 0 | 0 | 2 | 0 | 0 |
| 47 | 0 | 3 | 0 | 3 | 5 | 3 |
| 48 | 2 | 4 | 0 | 4 | 4 | 2 |
| 49 | 1 | 3 | 0 | 1 | 4 | 1 |
| 50 | 7 | 2 | 0 | 4 | 1 | 2 |
| Total # of cys | 200 | 217 | 15 | 204 | 206 | 93 |

| MELATONIN | Anterior Terminal | Anterior Intermediate | Anterior Proximal | Posterior Terminal |
|---|---|---|---|---|
| 1 | 7 | 9 | 0 | 8 |
| 2 | 6 | 8 | 0 | 6 |
| 3 | 4 | 4 | 0 | 6 |
| 4 | 7 | 4 | 1 | 8 |
| 5 | 0 | 0 | 0 | 6 |
| 6 | 5 | 2 | 0 | 6 |
| 7 | 4 | 5 | 1 | 5 |
| 8 | 4 | 2 | 0 | 5 |
| 9 | 3 | 7 | 0 | 6 |
| 10 | 6 | 8 | 1 | 5 |
| 11 | 4 | 1 | 0 | 6 |
| 12 | 2 | 2 | 0 | 4 |
| 13 | 7 | 5 | 3 | 8 |
| 14 | 6 | 12 | 0 | 7 |
| 15 | 5 | 3 | 0 | 6 |
| 16 | 7 | 8 | 0 | 7 |
| 17 | 3 | 4 | 0 | 6 |
| 18 | 0 | 8 | 5 | 5 |
| 19 | 5 | 7 | 0 | 4 |
| 20 | 3 | 7 | 0 | 5 |
| 21 | 3 | 6 | 0 | 5 |
| 22 | 4 | 4 | 0 | 4 |
| 23 | 4 | 6 | 1 | 5 |
| 24 | 2 | 5 | 0 | 4 |
| 25 | 3 | 3 | 0 | 4 |
| 26 | 4 | 3 | 0 | 7 |
| 27 | 5 | 7 | 0 | 1 |
| 28 | 0 | 0 | 0 | 0 |
| 29 | 4 | 2 | 0 | 5 |
| 30 | 0 | 0 | 0 | 0 |
| 31 | 6 | 7 | 1 | 2 |
| 32 | 2 | 0 | 0 | 0 |
| 33 | 3 | 0 | 0 | 4 |
| 34 | 1 | 1 | 0 | 0 |
| 35 | 3 | 2 | 0 | 2 |
| 36 | 0 | 0 | 0 | 2 |
| 37 | 0 | 0 | 0 | 0 |

| | | | | | |
|---|---|---|---|---|---|
| | 38 | 3 | 1 | 0 | 1 |
| | 39 | 0 | 0 | 0 | 0 |
| | 40 | 1 | 2 | 0 | 1 |
| | 41 | 0 | 0 | 0 | 0 |
| | 42 | 3 | 0 | 0 | 3 |
| | 43 | 4 | 1 | 0 | 0 |
| | 44 | 5 | 4 | 0 | 0 |
| | 45 | 6 | 2 | 0 | 4 |
| | 46 | 3 | 2 | 0 | 1 |
| | 47 | 4 | 2 | 0 | 3 |
| | 48 | 3 | 3 | 0 | 3 |
| | 49 | 2 | 1 | 0 | 4 |
| | 50 | 2 | 2 | 1 | 6 |
| 935 | Total # of cys | 168 | 172 | 14 | 190 |

| Posterior Intermediate | Posterior Proximal |
|---|---|
| 5 | 5 |
| 7 | 2 |
| 6 | 4 |
| 11 | 2 |
| 2 | 2 |
| 3 | 4 |
| 3 | 1 |
| 2 | 0 |
| 6 | 5 |
| 5 | 2 |
| 7 | 0 |
| 5 | 3 |
| 9 | 5 |
| 10 | 2 |
| 8 | 2 |
| 7 | 5 |
| 6 | 6 |
| 4 | 2 |
| 6 | 1 |
| 6 | 3 |
| 6 | 2 |
| 1 | 2 |
| 5 | 1 |
| 2 | 0 |
| 2 | 1 |
| 5 | 3 |
| 6 | 2 |
| 0 | 4 |
| 4 | 1 |
| 0 | 0 |
| 2 | 4 |
| 0 | 0 |
| 5 | 0 |
| 0 | 0 |
| 1 | 2 |
| 1 | 0 |
| 0 | 0 |

| | | |
|---|---|---|
| 3 | 0 | |
| 0 | 0 | |
| 0 | 0 | |
| 0 | 0 | |
| 3 | 1 | |
| 3 | 2 | |
| 2 | 3 | |
| 1 | 1 | |
| 6 | 2 | |
| 3 | 3 | |
| 4 | 0 | |
| 3 | 1 | |
| 2 | 0 | |
| 188 | 91 | 823 |
